# Stabilization Effects Induced by Trehalose on Creatine Aqueous Solutions Investigated by Infrared Spectroscopy

**DOI:** 10.3390/molecules27196310

**Published:** 2022-09-24

**Authors:** Maria Teresa Caccamo, Salvatore Magazù

**Affiliations:** 1 Dipartimento di Scienze Matematiche e Informatiche, Scienze Fisiche e Scienze Della Terra, Università degli Studi di Messina, Viale Ferdinando Stagno D’Alcontres 31, 98166 Messina, Italy; 2 Consorzio Interuniversitario Scienze Fisiche Applicate (CISFA), Viale Ferdinando Stagno D’Alcontres 31, 98166 Messina, Italy

**Keywords:** creatine, infrared spectroscopy, spectral distance, cross wavelet correlation

## Abstract

Creatine is a very popular amino acid widely utilized in the sports world due to its functions mainly related to muscle building and increasing performance. The present work investigates the behavior of creatine aqueous solutions and of creatine aqueous in the presence of trehalose as a function of time changes by means of Infrared spectroscopy. Infrared spectra have been gathered and studied over time for both the full spectrum and the intramolecular OH-stretching region for the two mixtures. This latter region was studied more specifically using a cutting-edge technique called Spectral Distance (SD). From this analysis of the spectral features of the investigated samples, it emerges that trehalose has a significant stabilizing effect on creatine aqueous solutions.

## 1. Introduction

Creatine or methylguanidino-acetic acid, whose chemical formula is C_4_H_9_N_3_O_2_, is an amino acid present in foods of animal origin (e.g., meat and fish) that is produced within our body and is currently available in powder and pills, especially for its widespread employment in the sports world [1,2,3].

The first person to successfully extract this molecule from meat was the French chemist Michel Eugène Chevreul, who discovered it in 1832. Because of this, he gave it the name creatine, which is derived from the Greek word “kreas,” which means meat. [4,5]. A few years later, in 1847, its presence was also confirmed by the German chemist Justus von Liebig, who had also observed a close link between this compound and muscle activity [6,7]. Creatine is in fact distributed throughout the body, where about 95% is in the skeletal muscles and about 5% is in the brain. The human body is able to synthesize it starting from three amino acids called glycine, methionine, and arginine. Creatine is produced in the liver, pancreas, and kidneys and then it is sent primarily to the muscles, brain, and heart [8,9].

Creatine is an essential substance, mainly for providing energy during muscle contraction. In the cell, the oxidation of carbohydrates and fats produces ATP, adenosine triphosphate, a molecule used to provide the energy necessary for the various cellular processes with the formation of ADP, adenosine diphosphate, and AMP, adenosine monophosphate. The main role of creatine, in the form of phosphocreatine, is to donate a phosphate group to ADP by converting it back into ATP, which can be used in a new cycle of reactions [10,11,12].

The amount of ATP present in the cell is very low and is rapidly exhausted. Creatine intervention allows one to maintain vigorous muscle contraction for a longer period of time without having the cell use oxygen [13,14,15].

After many years of studies and research, creatine has been given considerable importance in cellular energy metabolism, to the point of being used commercially as a supplement for athletes to improve sports performance and increase muscle mass [16,17,18,19].

The use of creatine in dietary supplements has become increasingly popular for improving muscle strength and athletic performance in short-term sports that require high physical exertion, such as running, swimming, and track cycling on short courses. Thanks to the action of creatine on muscle mass, it is also used in bodybuilding, rowing, and weightlifting. However, the use of creatine in dietary supplements has different effects depending on the muscle mass, the amount of creatine already present, and the amount introduced into the diet [20,21,22,23].

Creatine is often used in the treatment of many diseases, including fibromyalgia, Huntington’s disease, multiple sclerosis, and congestive heart failure, and the effects of creatine in the treatment of other diseases are being studied. Furthermore, it appears that creatine can help protect the health of patients at risk of ischemic heart disease or stroke and ensure the good development of the fetus during pregnancy, and it has an antioxidant action [24,25].

Trehalose (α-D-glucopyranosyl-α-D-glucopyranoside or alpha, alpha-trehalose, or α-D-glucopyranosyl-α-D-glucopyranoside, dihydrate) is a sugar, and more precisely a disaccharide (or disaccharide). Unlike sucrose, composed of a molecule of glucose linked to a molecule of fructose, trehalose is composed of two molecules of glucose linked together by an α-bond, α-1,1 (or “1,1-α- glycosidic”) particularly stable. Trehalose has the same chemical formula, C_12_H_22_O_11_, as the other two disaccharides, i.e., maltose and sucrose but different geometrical structures. It is a sugar, almost odorless, composed of white or almost white crystals, with a sweet taste [26,27,28,29].

It begins with a funny drug called Trehala (a soft cocoon formed by insects of the genus Larinus, living on different species of Echinops in the Middle East), used to treat coughs and lung pathologies, and presented at the International Exhibition of Paris, in 1855 by François Della Sudda. In 1858, Marcellin Berthelot (1827–1907) isolated trehalose from this atypical drug and reported its physical and chemical properties. In 1876, Müntz established the fact that trehalose, present in the cocoon, has the same chemical structure as the sugar isolated from Claviceps purpurea by Mitscherlich [30].

In 1913, trehalose was discovered for the first time in the plant environment and, more specifically, in Selaginella lepidophylla. Trehalose will subsequently be found in mosses, green algae, ferns, and liverworts and in a very small number of angiosperms, characterized by their resistance to desiccation. Trehalose was occasionally detected in blooming plants, but the levels were often very small and were thought to be microbial or fungal in origin [31,32]. In the plant environment, genes coding for active trehalose-phosphate-synthetase and for trehalose-phosphatases were identified at the end of the 1990s in Arabidopsis thaliana, a Brassicassaceae corresponding to the attractive French name Arabette des dames. Osmolyte, protector against stress, carrier of carbon, trehalose often gives way in the plant environment to sucrose, giving it primacy (the latter is often 100 to 1000 times better represented than trehalose) [33].

In the 1970s, Clegg studied brine shrimp (Artemia salina) and showed the importance of trehalose in the resistance of encysted embryos to desiccation [34,35]. In a state of anhydrobiosis (a slow state of life linked to low water content), cysts can survive for several decades [36].

Trehalose presents physical–chemical properties different from the other homologous disaccharides, which make it used not only in the food industry but also in pharmaceuticals and cosmetics, as well as in “energy” drinks or foods for athletes, as an additive protective agent for baker’s yeasts, or as a rust inhibitor [37,38,39,40,41,42].

The present work focused on explaining the key role that trehalose plays in bioprotective and shelf-life mechanisms [43,44,45,46,47,48].

For this purpose, according to several researchers [49,50,51], the features of the solvent have a major influence on the dynamics of proteins, and as a result, water/bioprotectant molecule combinations have received much attention.

Within this context, the higher glass transition temperature (T_g_) of trehalose and its mixtures with water, in comparison to the other disaccharides, is the only explanation for its superior bioprotectant effectiveness, according to Green and Angell [52]. The higher T_g_ values of the trehalose/H_2_O mixtures, in comparison to the Tg values of the other disaccharides/H_2_O mixtures at all concentration values, imply that at a given temperature, the glass transition for trehalose and its mixtures with water always occurs at a higher water content. This trait is crucial for bioprotection.

However, this explanation is not sufficient on its own because there are other related systems, such as dextran ((C_6_H_10_O_5_)x) [51], a linear polysaccharide with α(1–6) glycosidic linkages, which has a greater T_g_ value but does not exhibit a similar bioprotective effect.

On the other hand, the “water-replacement hypothesis” was developed by Crowe et al. [53] to explain the protective function of trehalose by trehalose’s direct hydrogen bonding with polar headgroups of the lipids, just as water does. In contrast, Crowe et al. hypothesized that a direct interaction between the sugars and the biostructures occurs.

Numerous experimental results from various spectroscopic examinations, together with certain computer studies, unmistakably show that disaccharides and trehalose have the greatest impact on the structural and dynamical features of water [44,45,46].

More specifically, the destruction of the tetrahedral coordination of pure water is demonstrated by neutron diffraction results, which demonstrate for all disaccharides a strong distortion of the peaks linked to the hydrogen-bonded network in the partial radial distribution functions.

The addition of trehalose, in contrast to the other disaccharides, breaks down the tetrahedral intermolecular network of water, which results in the formation of ice by a decrease in temperature, according to Raman scattering data. These findings demonstrate the distinct kosmotropic nature of disaccharides by demonstrating that the intensity of the contact between the disaccharide and the water molecule is greater than that between the water molecules. Additionally, ultrasonic velocity tests provide evidence that, in comparison to the other disaccharides, the trehalose–water system exhibits the highest solute–solvent interaction strength and hydration number across all concentration ranges.

In terms of dynamics, Quasi Elastic Neutron Scattering (QENS) findings on disaccharide solutions show that trehalose, in particular, has a significant impact on water dynamics when disaccharides are present. Additionally, trehalose has a stronger kinetic character in the Angell’s classification system than the other disaccharides, according to viscosity studies on trehalose, maltose, and sucrose aqueous solutions. The low-frequency dynamics of trehalose, maltose, and sucrose water mixtures across the glass transition were further studied using QENS and Inelastic Neutron Scattering (INS). The results of the experiments demonstrate that the trehalose/H_2_O mixture has a stronger character, as seen by the relaxational to vibrational contribution ratio [54].

The degree of fragility of glass-forming systems has also been given a new operational definition [55], and in this context, the stronger nature of the trehalose/H_2_O mixture suggests a better attitude toward the ability of maltose and sucrose/H_2_O mixtures to encapsulate biostructures in a more rigid matrix.

Finally, it is noteworthy to stress that trehalose increases the surface tension and stabilizes the aqueous solution of creatine, preventing evaporation [56,57].

More precisely, creatine aqueous solutions and creatine aqueous solutions in the presence of trehalose have been studied as a function of time by means of infrared spectroscopy [58].

The class of spectroscopy known as infrared absorption (IR) focuses on the electromagnetic spectrum’s infrared region. It can be used, similar to certain other spectroscopic techniques, to identify compounds, establish the composition of a sample, and analyze changes in a composition of a sample with temperature or over time. It is well known that the infrared region of the electromagnetic spectrum is split into three groups: near, mid, and far infrared, named in connection with the visible spectrum [59,60,61]. The far infrared, which is adjacent to the microwave zone and ranges from roughly 400 to 10 cm^−1^ (1000–30 μm), has low energy and can be used for rotational spectroscopy. It is possible to examine fundamental vibrations and related rovibrational structures in the mid-infrared, which has a wavelength range of roughly 4000 to 400 cm^−1^ (30–1.4 μm). Harmonic vibrations can be stimulated by the more intense near-infrared, which falls between 14,000 and 4000 cm^−1^ (1.4–0.8 μm). The names and classifications of these sub-regions are fundamentally conventions. They are not based on precise chemical or electromagnetic properties, tight divisions, or other criteria [62,63].

The fact that molecules have distinctive frequencies at which they rotate or vibrate in relation to distinct energy levels is taken advantage of by infrared spectroscopy (vibrational modes). The atomic masses, the related vibronic coupling, and the form of the potential energy surfaces all influence these resonant frequencies. A molecular vibrational mode must be connected to modifications in the permanent dipole in order for it to be active in the infrared. In particular, within the Born–Oppenheimer and harmonic approximations, the resonant frequencies are determined by the normal modes corresponding to the potential energy surface of the molecular electronic ground state when the molecular Hamiltonian, corresponding to the electronic ground state, can be approximated by a harmonic oscillator in the vicinity of the equilibrium molecular geometry [64,65]. However, the resonance frequencies may initially be connected to the atomic masses of termination and the strength of the bond. Consequently, the vibration frequency can be connected to a specific bond. Since complex compounds frequently contain bonds, it is possible for vibrations to couple together, resulting in infrared absorptions at distinctive frequencies that can be coupled to chemical groups. A beam of infrared light is sent through a sample to produce the sample’s infrared spectrum. The quantity of energy absorbed at each wavelength can be determined by analyzing the transmitted light. A monochromatic beam, a change in wavelength over time, or a Fourier transform instrument that measures all wave measurements simultaneously can all be used to achieve this. Thus, the spectra in transmittance or in absorbance can be created. These features can be analyzed to learn more about the sample’s molecular makeup and how it has changed over time [66,67,68,69].

The Spectral Distance (SD) is a novel method for comparing spectra and characterizing sample changes as a function of time, which is used in the current study, as far as data processing is concerned [70,71].

## 2. Materials and Experimental Set-Up

Creatine aqueous solutions and creatine aqueous solutions in the presence of trehalose were prepared starting from pure creatine powder and pure trehalose, both purchased from Aldrich-Chemie, and double distilled water. For the mixtures, the investigated concentration values correspond to 90% of creatine and 10% of double distilled water for the binary system and to 90% of creatine, 8% of double distilled water, and 2% of trehalose for the ternary system. Concerning the data acquisition, the Fourier transform IR spectroscopy spectra in attenuated total reflection mode (ATR), were collected on samples brought into contact with the detection device element (crystal) and were performed in air. More specifically, the measurements were carried out on a Bruker alpha spectrometer equipped with an ATR stage with a diamond crystal, controlled by the OpusLab v 7.5 software. Here, the infrared beam is reflected on the internal surface of the crystal and creates an evanescent wave. Part of the energy of this wave is then absorbed, the reflected radiation being sent back to the detector [72]. One of the major advantages of this technique is the possibility of analyzing samples without special preparation. The acquisitions were carried out by performing 32 scans between 4000 and 400 cm^−1^, with a resolution of 0.4 cm^−1^.

## 3. Methods

The Spectral Distance (*SD*) is mathematically represented by the following expression:

(1) $$SD=\sqrt{I\left(\omega ,t_{0}\right)-I\left(\omega ,t\right)\cdot \Delta \omega }$$

where $I\left(\omega ,t_{0}\right)$ is the intensity at frequency ω and at time t=0 while Δω is the instrumental resolution.

When applied to different spectra, it furnishes a unique parameter to estimate the similarity degree between spectra acquired under different conditions or referring to different systems. More specifically, in our case, the *SD* measures the affinity between the reference spectrum, which is the spectrum acquired at time *t* = 0, and the spectra collected at different times. Such evaluation has been performed for the spectra of the two investigated systems [73,74,75,76]. Such a method allows one to characterize the system changes in time.

## 4. Results and Discussion

**Figure 1:**
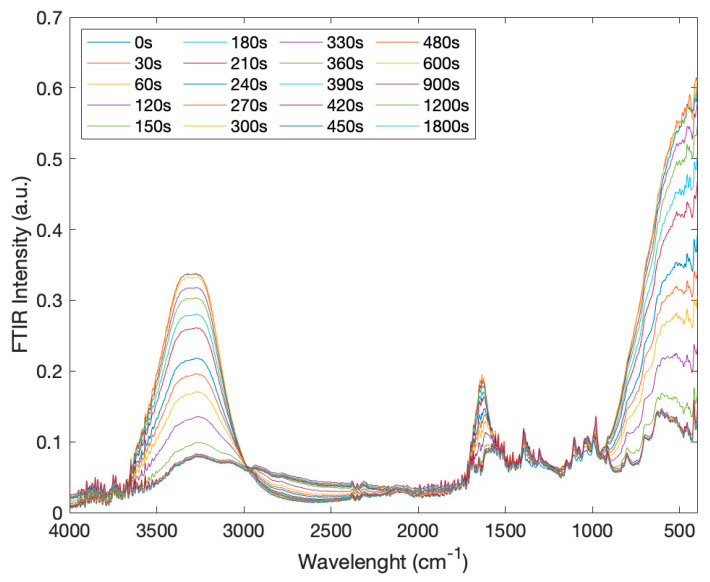
IR Spectra of creatine aqueous solutions as a function of time (range: 0 ÷ 1800 s) in the spectral range 4000–400 cm^−1^.

Figure 1 reports the infrared spectra of creatine aqueous solutions, in the spectral range of 4000–400 cm^−1^, as a function of time in the range of 0 ÷ 1800 s.

**Figure 2:**
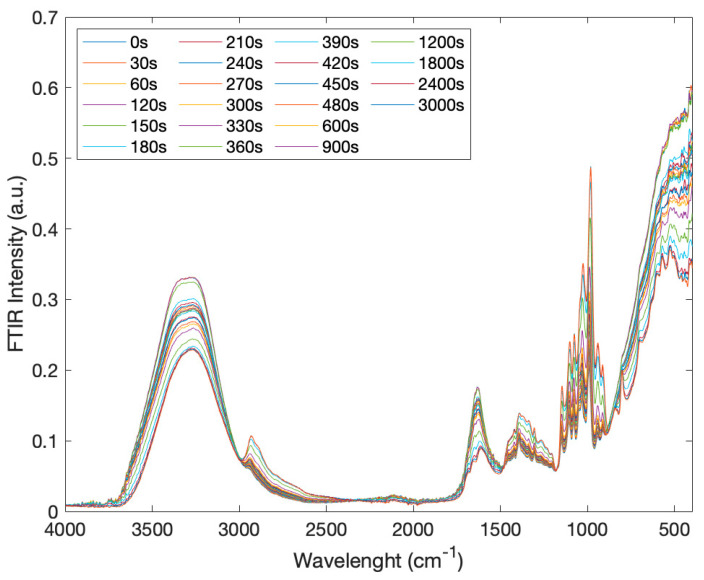
IR Spectra of creatine aqueous solutions in the presence of trehalose as a function of time (0 ÷ 3000 s) in the spectral range of 4000–400 cm^−1^.

Figure 2 reports the infrared spectra of creatine aqueous solutions in the presence of trehalose, in the spectral range of 4000–400 cm^−1^, as a function of time in the range of 0 ÷ 3000 s.

### 4.1. Infrared Spectra Integrated Area

**Figure 3:**
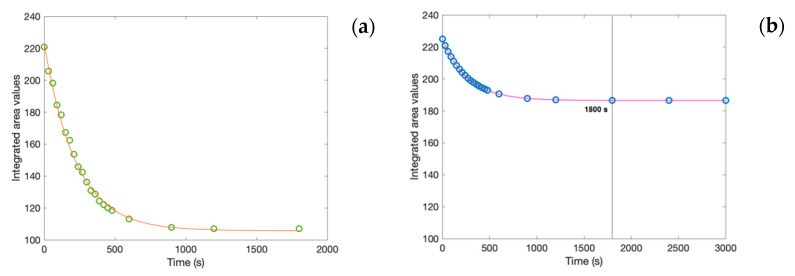
Integrated area values of the whole investigated spectra as a function of time for: (**a**) creatine aqueous solution; (**b**) creatine aqueous solution in the presence of trehalose.

In order to detect behavioral differences between the binary and the ternary investigated systems, the spectra integrated areas were calculated for the whole spectral range (i.e., 4000 to 400 cm^−1^). Figure 3a (on the left) reports the calculated integrated area values of the whole investigated spectra as a function of time for the creatine aqueous solution, while Figure 3b (on the right) shows the integrated area values of the whole investigated spectra versus time for the creatine aqueous solution in the presence of trehalose.

As it can be seen from the inspection of the figures, the calculated integrated area values fulfill a decaying exponential law reaching a plateau value in the long-time range. In particular, the decaying characteristic time, which is 267 s for the ternary system and 220 s for the binary system, and the plateau value, which is 190 for the ternary system and 100 for the binary system, in the presence of trehalose is higher with respect to the binary system. These results suggest that the presence of trehalose significantly stabilizes in time with the creatine solution. Furthermore, the black line in Figure 3b indicates the comparison of the same time between the binary system and the ternary system.

### 4.2. OH-Stretching Integrated Area

Since a significant contribution to the total integrated area is derived from the intramolecular OH-stretching region, in the following, we focused the attention on the spectral feature changes as a function of time in the restricted region 2800–3700 cm^−1^.

**Figure 4:**
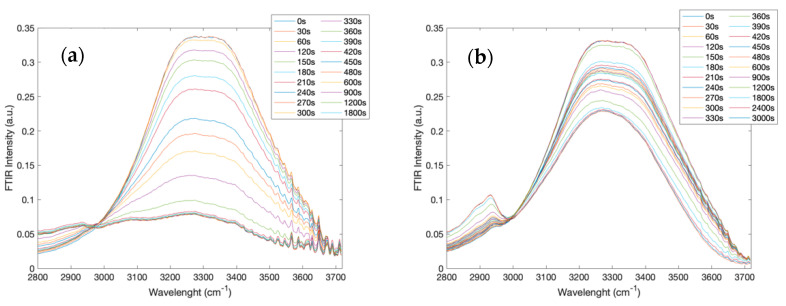
Infrared spectra at different times in the 2800–3700 cm^−1^ spectral range, corresponding to the intramolecular OH-stretching region, for the creatine aqueous solution (**a**) and for the creatine aqueous solution in the presence of trehalose (**b**).

Figure 4 reports the intramolecular OH-stretching spectra at different times in the 2800–3700 cm^−1^ spectral range for the creatine aqueous solution (a) and for the creatine aqueous solution in the presence of trehalose (b).

What emerges from the comparison of the two data sets is that the intramolecular OH-stretching mode revealed a marked dependence on time for the creatine aqueous solution, while the addition of trehalose to the binary solution reduced such a dependence.

**Figure 5:**
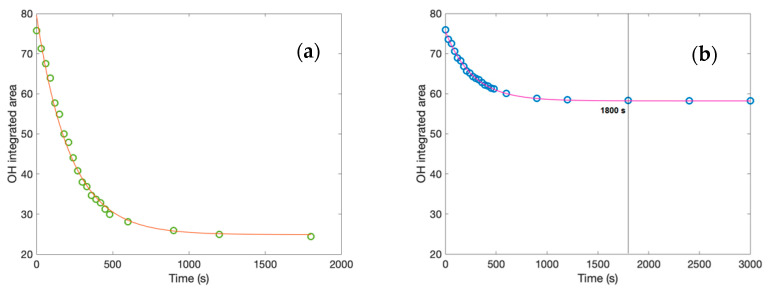
Integrated area values of the OH-stretching region, i.e., 2800–3700 cm^−1^, for all the investigated spectra as a function of time in the spectral range 2800–3700 cm^−1^ for: (**a**) creatine aqueous solution; (**b**) creatine aqueous solution in the presence of trehalose.

In order to detect quantitative differences between the binary and the ternary investigated systems, the spectra integrated areas have been calculated for the spectral range 2800–3700 cm^−1^. Figure 5a (on the left) reports the calculated integrated area values of the OH-stretching region, i.e., 2800–3700 cm^−1^, for all the investigated spectra as a function of time for the creatine aqueous solution; while Figure 5b (on the right) shows the integrated area values of the OH-stretching region versus time for the creatine aqueous solution in the presence of trehalose [42,44].

As it can be seen, the calculated integrated area values of the OH-stretching region fulfill a decaying exponential law reaching a plateau value in the long-time range. In particular, the decaying characteristic time, which is 270 s for the ternary system and 220 s for the binary system, and the plateau value, which is 190 for the ternary system and 100 for the binary system, in the presence of trehalose is higher with respect to the binary system. These results are in agreement with the data obtained considering the total spectral range (i.e., 4000 to 400 cm^−1^) and confirm that the presence of trehalose significantly stabilizes in time with the creatine solution [45,60]. In addition, in this case, the black line in Figure 5b specifies the comparison of the same time between the binary system and the ternary system.

### 4.3. Spectral Distance Analysis of Normalized Spectra

**Figure 6:**
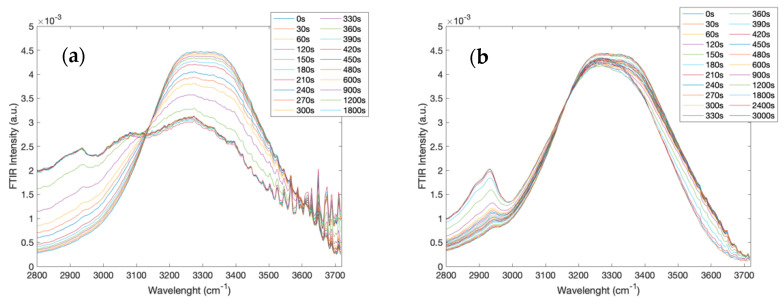
Normalized spectra in the intramolecular OH-stretching region, i.e., in the spectral range of 2800–3700 cm^−1^, for the creatine aqueous solution (**a**) and for the creatine aqueous solution in the presence of trehalose on (**b**).

Figure 6 reports the normalized spectra relative to the intramolecular OH-stretching region, i.e., in the spectral range of 2800–3700 cm^−1^, for the creatine aqueous solution on the left and for the creatine aqueous solution in the presence of trehalose on the right.

**Figure 7:**
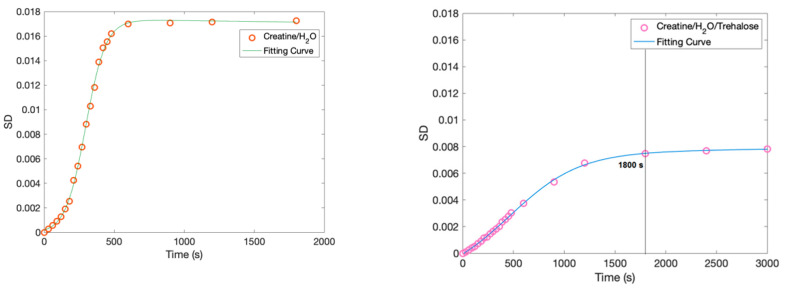
SD calculated values versus time for the creatine aqueous solution on the (**left**) and for the creatine aqueous solution in the presence of trehalose on the (**right**).

In order to better clarify the role played by trehalose, in the following, we apply the Spectral Distance analysis, taking as the reference spectrum the one at time 0. Figure 7 shows the values of the Spectral Distance calculated, by means of Equation (1), for the creatine aqueous solution on the left and for the creatine aqueous solution in the presence of trehalose on the right [53,64].

As it can be seen, the SD values versus time fulfill an increasing sigmoid law reaching a plateau value in the long-time range only for both systems investigated. However, in the case of the creatine aqueous solution in the presence of trehalose, the incremental rate is much lower with respect to the binary system. This result is in agreement with the previous findings and confirms that the presence of trehalose stabilizes significantly in time with the creatine solution.

## 5. Conclusions

The goal of the current work is to employ infrared spectroscopy to investigate how creatine aqueous solutions and creatine aqueous solutions, in the presence of trehalose, behave as a function of time.

Both the whole infrared spectra and the intramolecular OH-stretching bands have been collected and examined as a function of time for the two investigated systems, and the latter spectral region was investigated by using the supposed Spectral Distance (SD) approach. Such a procedure, applied to different spectra, furnishes a unique parameter to estimate the similarity degree between spectra acquired under different conditions allowing one to characterize the system changes in time

According to the obtained experimental findings and the performed data analyses, trehalose has a substantial stabilizing impact on creatine aqueous solutions.

## Data Availability

Not applicable.
